# Quasicriticality explains variability of human neural dynamics across life span

**DOI:** 10.3389/fncom.2022.1037550

**Published:** 2022-11-30

**Authors:** Leandro J. Fosque, Abolfazl Alipour, Marzieh Zare, Rashid V. Williams-García, John M. Beggs, Gerardo Ortiz

**Affiliations:** ^1^Department of Physics, Indiana University, Bloomington, IN, United States; ^2^Department of Psychological and Brain Sciences, Indiana University, Bloomington, IN, United States; ^3^AI Practice, CGI, Montreal, QC, Canada; ^4^Département de Physique, Institut Denis Poisson (UMR7013), Tours, France

**Keywords:** magnetoencephalography (MEG), quasicriticality, Cam-CAN dataset, healthy human, aging, neuronal avalanches

## Abstract

Aging impacts the brain's structural and functional organization and over time leads to various disorders, such as Alzheimer's disease and cognitive impairment. The process also impacts sensory function, bringing about a general slowing in various perceptual and cognitive functions. Here, we analyze the Cambridge Centre for Ageing and Neuroscience (Cam-CAN) resting-state magnetoencephalography (MEG) dataset—the largest aging cohort available—in light of the quasicriticality framework, a novel organizing principle for brain functionality which relates information processing and scaling properties of brain activity to brain connectivity and stimulus. Examination of the data using this framework reveals interesting correlations with age and gender of test subjects. Using simulated data as verification, our results suggest a link between changes to brain connectivity due to aging and increased dynamical fluctuations of neuronal firing rates. Our findings suggest a platform to develop biomarkers of neurological health.

## 1. Introduction

It is extremely useful in medicine to have biomarkers that will diagnose or predict a patient's health (Montez et al., 2009; Zijlmans et al., 2012; Mena et al., 2016; Bruining et al., 2020). In neuroscience, the rapid advancement of data collection and analysis has generated many new candidate biomarkers, like in seizure prediction, for example (Scheffer et al., 2009; Kuhlmann et al., 2018). However, it is sometimes difficult to tell if a new biomarker underlies the cause of a condition or merely has a spurious correlation with it (Granger and Newbold, 1974; Mormann et al., 2007; Calude and Longo, 2017). A strategy to avoid this pitfall would be to focus on observables that are tied to homeostatic functions that are known to establish conditions for health.

A growing body of work suggests that the brain homeostatically regulates itself to operate near a critical point where information processing is optimal (Meisel et al., 2017; Ma et al., 2019; Beggs, 2022). At this critical point, incoming activity is neither amplified (supercritical) nor damped (subcritical), but approximately preserved as it passes through neural networks (Beggs, 2008). Departures from the critical point have been associated with conditions of poor neurological health like epilepsy (Meisel et al., 2012), Alzheimer's disease (Montez et al., 2009) and depression (Gärtner et al., 2017; for a review, see Zimmern, 2020). For example, when subjects are deprived of sleep, their brain signals as assessed by electroencephalography (EEG) move from near the critical point toward being supercritical, where there is an increased likelihood of seizures. After restorative sleep, subjects move back toward being subcritical where seizures are less likely (Meisel et al., 2013). Homeostasis of criticality has been observed in animal experiments too, where prolonged eye suture causes visual cortex to become subcritical. After several days under this condition, the cortex returns toward the critical point (Ma et al., 2019). These and other studies (Tetzlaff et al., 2010; Shew et al., 2015; Fontenele et al., 2019) indicate that there are mechanisms to maintain the brain near the critical point, even in the face of strong perturbations.

The brain, however, is never exactly critical and it is natural to ask if there is an organizing principle behind the neocortex's functional behavior. The quasicriticality framework (Williams-Garćıa et al., 2014) has been advanced as such a principle. A new way to plot the brain's proximity to the critical point, rooted in this idea of quasicriticality, has revealed that the effective critical exponents (explained more below) are confined to lie near a scaling line (Friedman et al., 2012). For example, as a rat explores, grooms, or sleeps over several hours, its effective critical exponents may change, but they consistently lie near this scaling line, moving along it over time (Fontenele et al., 2019). In another experiment that illustrates this, as rats learned a lever pressing task over several weeks their effective critical exponents moved along the scaling line but never strayed far from it (Ma et al., 2020). Similar findings have now been reported by several labs, including our own (Shew et al., 2015; Fosque et al., 2021). This makes it reasonable to ask if a new biomarker for neurological health could be based on the brain's tendency to operate within the quasicritical region. Based on this work, we hypothesize that the position, and its change, along this scaling line could serve as a biomarker for neurological health.

To pursue this, we examined a large set of magneto-encephalography (MEG) data collected from 604 healthy human subjects. We extracted the effective critical exponents from each patient and plotted them along the scaling line. As a first step toward testing this idea, we asked if the position on the scaling line contained information about the patients' age, gender, and sensitivity to inputs. We found that statistically significant relationships did exist, suggesting that this approach will be fruitful for other health-related information.

To quantitatively interpret these results, we used a previously-published computational model of brain dynamics, the so-called *cortical branching model* (CBM) (Williams-Garćıa et al., 2014). When we supplied this model with activity levels and connectivity patterns that approximated those from the patient population, it produced outputs that were consistent with the results from the human data. This model gives us an intuitive understanding of why the effective critical exponents move along the scaling line and how they might be related to departures from criticality.

The remainder of this paper is organized as follows. In the next section we will briefly explain some background information related to quasicritical behavior, like effective critical exponents, the scaling relation and scaling line, as some of these concepts are imported from physics and are not widely known in neuroscience. After that, we will describe the methods of data collection and analysis. This will be followed by the results section, which describes the data analysis and computational modeling. Lastly, we will discuss possible limitations and future applications of this approach.

## 2. Background: The organizing principle of quasicriticality

Broadly speaking, activity in neural networks propagates in successive stages, spreading from one set of active neurons to another, and resulting in spatio-temporal patterns of activation known as neuronal avalanches. This propagation can be quantified using the branching ratio σ, which gives the average number of neurons activated by a single active neuron. If we consider a network with infinitesimal inputs, then the network is supercritical when σ>1, and incoming signals are successively amplified. After several stages, it is difficult to discern from the output which neurons were active at the input because the network becomes nearly saturated with activity. When σ < 1, the network is subcritical and incoming signals are damped. After several stages, it is also difficult to discern which neurons were active at the input, now because there is almost no activity at the output. When σ≈1, the network nears a critical point where levels of activity are roughly maintained. In this condition, it is easiest to use the output to reconstruct the input; mutual information between inputs and outputs is maximized for critical systems (Shew et al., 2011). For similar reasons, a network's dynamical response to inputs, i.e., its susceptibility, is also maximized near the critical point and diverges at the critical point σ = 1. It has become clear that this picture seems consistent with experimental evidence (Shew et al., 2011; Wilting and Priesemann, 2019). This type of evidence has been obtained from *in vitro* culture preparations (Beggs and Plenz, 2003) as well as *in vivo* recordings from fish (Ponce-Alvarez et al., 2018), rodents (Fontenele et al., 2019), primates (Petermann et al., 2009), and humans (Tagliazucchi et al., 2012; Shriki et al., 2013).

**Figure 1:**
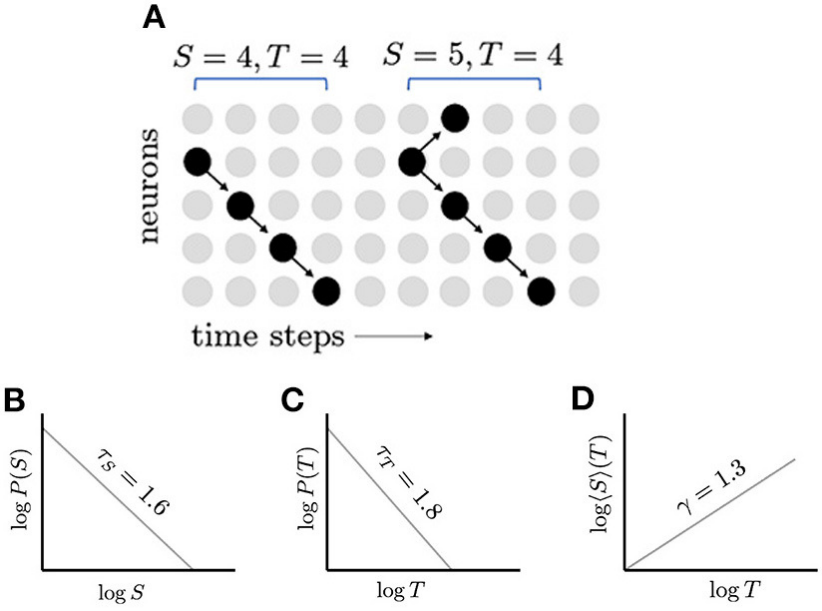
Neuronal avalanches and their distributions. **(A)** Schematic network of five neurons; inactive neurons are represented by gray dots and active neurons by black dots; and propagation of activity is represented by arrows. Two avalanches are shown: the first with size 4 and duration 4, and the second with size 5 and duration 4. **(B)** In a critical network, the distribution of avalanche sizes *S*, follows a power law, which appears linear when plotted with logarithmic scales. The slope of this line gives the exponent τ_*S*_ = 1.6. **(C)** The distribution of avalanche durations, *T*, follows another power law with exponent τ_*T*_ = 1.8. **(D)** The average avalanche size for a given duration, 〈*S*〉(*T*), follows a power law with exponent γ = 4/3. The appearance of multiple power laws and the relationship between their exponents, (τ_*T*_−1)/(τ_*S*_−1) = γ, indicate that the network may be operating near a critical point.

Other signatures of criticality include the scale-free distributions of measured quantities, for example, the size and duration of neuronal avalanches. At criticality, probability distributions of avalanche quantities *q* (e.g., avalanche size *S* and duration *T*) conform to the finite-size scaling assumption, $P\left(q,L\right)=q^{-\tau_{q}}{\text{Ψ}}_{q}\left(q/L^{d_{q}}\right)$, that establishes relations between the critical exponents τ_*q*_ and fractional dimension *d*_*q*_, given a system of linear size *L*, and where Ψ_*q*_ is the scaling function (Nishimori and Ortiz, 2011). Close to criticality, avalanche size and duration distributions nearly follow power laws, showing approximate scale invariance (Figure 1). The fact that these distributions are nearly scale-free indicates the absence of a dominant length or time scale, which would be small in the case of subcritical networks and large in the case of supercritical networks. Relations between the characteristic exponents of these power laws help to confirm critical behavior. For instance, as indicated above, the network produces cascades, or avalanches, of activity whose sizes *S* and durations *T* follow power law distributions, $P\left(S\right)\propto S^{-\tau_{S}}$ and $P\left(T\right)\propto T^{-\tau_{T}}$, respectively. When represented in a log-log plot, power law distributions appear as straight lines and the slopes of these distributions are used to estimate critical exponents. When the average avalanche size 〈*S*〉 is plotted against avalanche duration, this also produces a straight line in a log-log plot; the exponent for this is γ, and the distribution 〈*S*〉∝*T*^γ^. At criticality, the avalanche size exponent τ_*S*_ and avalanche duration exponent τ_*T*_ are related by the exact scaling relation (Jensen, 1998; Sethna et al., 2001; Henkel et al., 2008; Friedman et al., 2012),


(1) $$\begin{array}{l} \gamma =\frac{\tau_{T}-1}{\tau_{S}-1}, \end{array}$$

where γ corresponds to the characteristic exponent of average avalanche size for a given duration, 〈*S*〉(*T*), another scale-free quantity. The relations above are only *exactly* satisfied at criticality. At criticality, universality dictates a unique set of critical exponents and scaling relations among them. These relations will not be satisfied away from the critical point, complicating the general problem of quantifying closeness to criticality in non-equilibrium systems. Recently, some attempts have been done in this direction, but there is still much work to be made (Palmieri and Jensen, 2020a,b).

Interestingly, in living systems, pairs of exponents (τ_*T*_, τ_*S*_) seem to *approximately* satisfy the scaling relation (1), with pairs of exponents organized around the scaling line. Then, one wonders whether there is a sense in which one can assess proximity to a critical point(s): (1) Is it possible to determine quantitatively how close a neural network can be to its optimal response, i.e., largest dynamical susceptibility? And, most importantly, (2) Is there an organizing principle for neural dynamics?

The quasicriticality hypothesis states that living neural systems, being constantly bombarded by external input, will adapt to operate in the functional parameter region near the peak of maximum dynamical susceptibility of neural activity (Williams-Garćıa et al., 2014). This quasicritical region maximizes information propagation across the neural network. Moreover, the peak of maximum susceptibility defines a non-equilibrium Widom line (or surface) that depends on the level of external noise (or stimulus) as well as other functional parameters of the network. It is important to emphasize that the quasicritical hypothesis represents a universal organizing principle and not a particular non-equilibrium model of brain cortex dynamics (such as the CBM, Williams-Garćıa et al., 2014). As mentioned earlier, there is now evidence of homeostasis toward a *quasicritical* region, even after strong disruptions. In this sense, homeostasis of neuronal activity is analogous to homeostasis of blood pressure, heart rate, and body temperature. Because it is useful to track these vital signs over time, it would also make sense to track proximity to this quasicritical region over time. A new way to do this has emerged from the previously-mentioned exponent relation and the organizing principle of quasicriticality (Williams-Garćıa et al., 2014; Fontenele et al., 2019; Fosque et al., 2021). In this framework, although the network is not exactly at the critical point, it may still have approximate power-law distributions with *effective critical exponents*. When the network operates away from the critical point, the two sides of Equation (1) will not *exactly* equal each other. The magnitude of this difference has been called the distance to criticality coefficient (DCC; Ma et al., 2019). At, or near, the Widom line this relation will be approximately satisfied (Williams-Garćıa et al., 2014; Fosque et al., 2021) providing evidence for quasicritical behavior.

**Figure 2:**
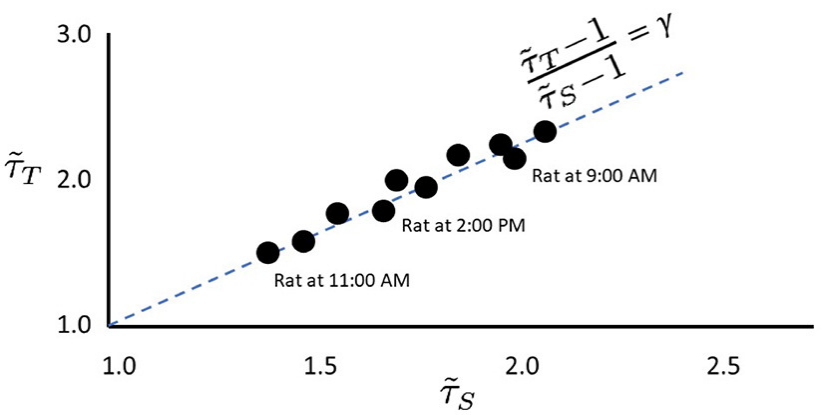
Empirical data stay near the γ-scaling line. Depiction of avalanche duration exponents, ${\tilde{\tau }}_{T}$, plotted against avalanche size exponents, ${\tilde{\tau }}_{S}$ for data collected from the same rat at different times (similar to data presented by Fontenele et al., 2019). Note that in all cases, exponents lie close to the dashed γ-scaling line given by $\gamma =\left({\tilde{\tau }}_{T}-1\right)/\left({\tilde{\tau }}_{S}-1\right)$. Proximity to the γ-scaling line indicates closeness to the critical point.

The use of scaling exponents and the relations among them is based upon previous works (Williams-Garćıa et al., 2014; Fosque et al., 2021) which showed that when the system's response, subject to noise, is near the peak of maximum dynamical susceptibility, it maximizes the region of scale-invariance in avalanche distributions with a scaling relation closest to being satisfied. The effective avalanche duration exponent, ${\tilde{\tau }}_{T}$, and the effective avalanche size exponent, ${\tilde{\tau }}_{S}$ can be plotted as a point in the $\left({\tilde{\tau }}_{T},{\tilde{\tau }}_{S}\right)$ plane. Over time, depending on stimuli, noise, and intrinsic structural parameters of the neural network, it is found that while ${\tilde{\tau }}_{T}$ and ${\tilde{\tau }}_{S}$ may change, they typically remain close to the so-called γ-scaling line, given by the exact scaling relation in Equation (1). This is shown schematically in Figure 2. The fact that the same system under different stimuli or noise displays different pairs of effective exponents is an indication that the dynamics of that system *cannot be critical*. We argue that the hypothesis of criticality needs to be replaced by the hypothesis of quasicriticality.

**Figure 3:**
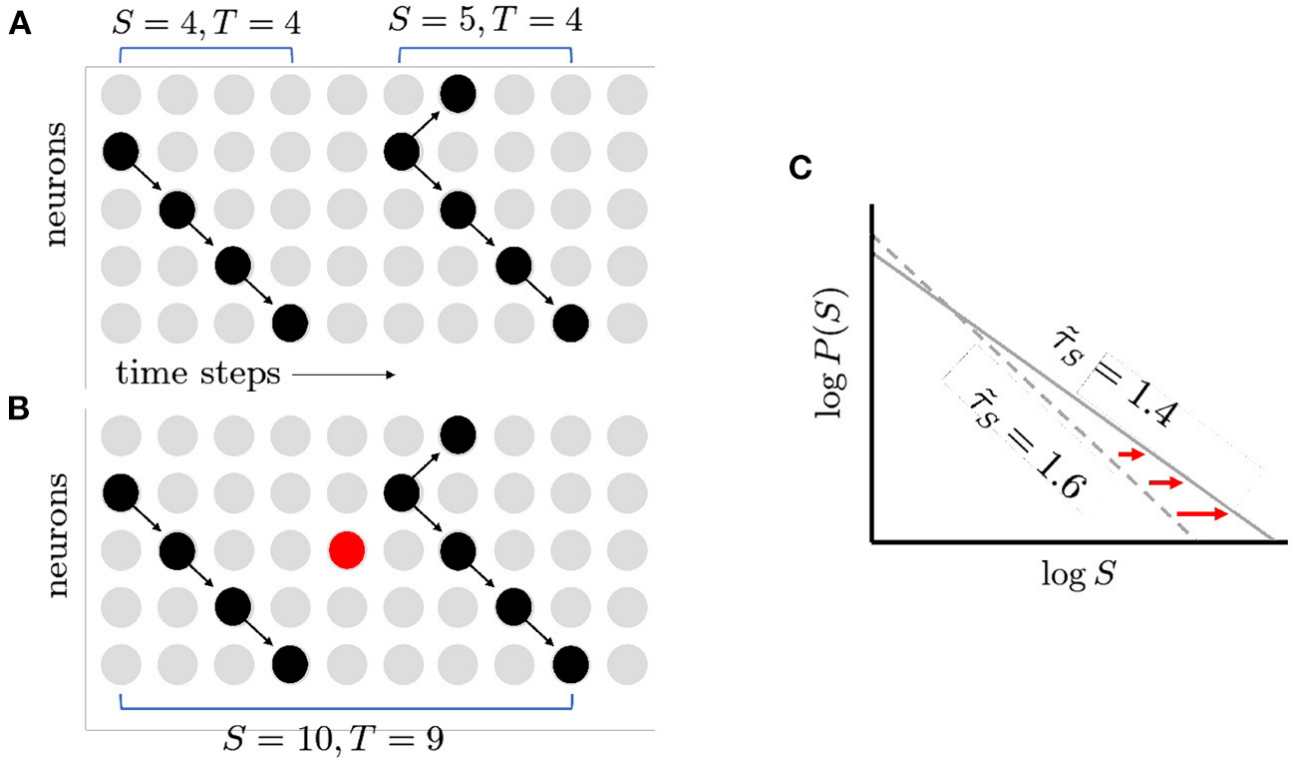
How spontaneous activity reduces exponent magnitude. **(A)** Two avalanches as shown before. Note gap in activity separating them. **(B)** Spontaneous activity turns on neurons that would otherwise have been inactive (red circle), filling the gap. The two previously distinct avalanches are now merged together into a larger and longer avalanche. **(C)** The increase in large avalanches causes the tail of the distribution to move further to the right. This in turn causes the magnitude of the exponent ${\tilde{\tau }}_{S}$ to decrease (1.6–1.4). The figure illustrates schematically the results of quasicritical simulations (Fosque et al., 2021).

**Figure 4:**
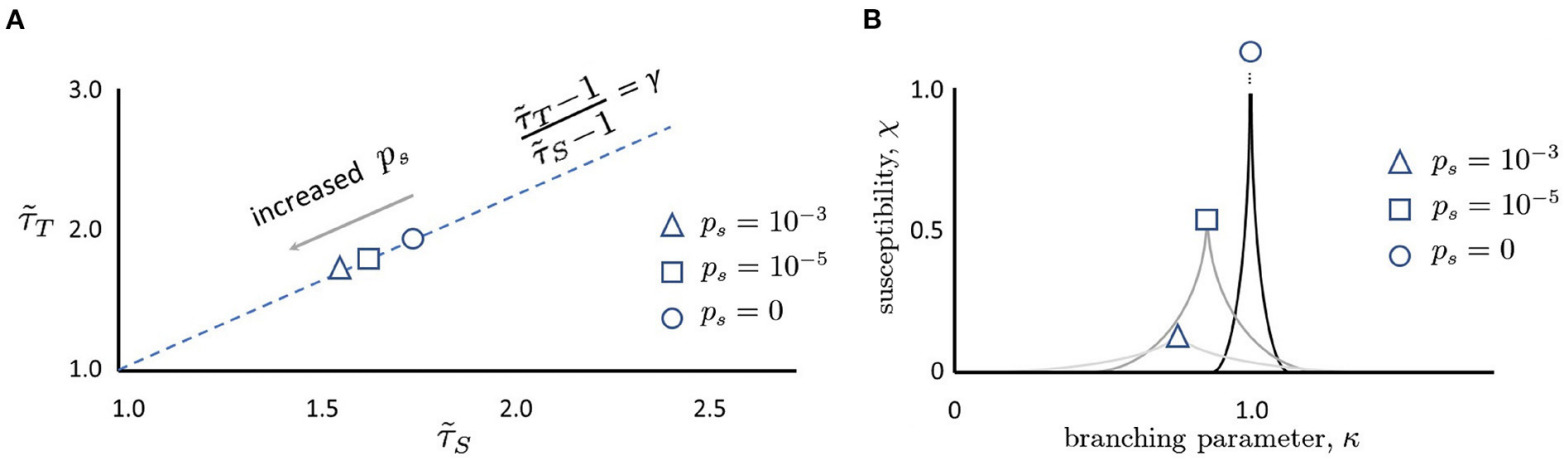
**(A)** Moving along the γ-scaling line. Avalanche duration exponents, ${\tilde{\tau }}_{T}$, plotted against avalanche size exponents, ${\tilde{\tau }}_{S}$ for different values of *p*_*s*_. Note that in all cases, exponents lie close to the dashed γ-scaling line given by the equation $\gamma =\left({\tilde{\tau }}_{T}-1\right)/\left({\tilde{\tau }}_{S}-1\right)$. When there is no spontaneous activity, the magnitude of the exponents is largest (circle). As *p*_*s*_ is increased, the magnitude of the exponents decreases (square, then triangle). Schematic plot here summarizes results reported by Shew et al. (2015), Fontenele et al. (2019), and Fosque et al. (2021), and predicted by models of quasicriticality (Williams-Garćıa et al., 2014; Fosque et al., 2021). **(B)** Susceptibility, χ, is blunted by increases in *p*_*s*_. When *p*_*s*_ = 0 and the branching parameter is exactly one, the susceptibility curve will diverge to infinity (circle). As *p*_*s*_ is increased (square, triangle), the susceptibility declines. Note that the branching parameter at which these curves peak also declines. This figure schematically depicts the predictions of a quasicritical network model (Williams-Garćıa et al., 2014) that have recently been corroborated with data from spiking networks (Fosque et al., 2021).

What things can push a network away from the critical point? Randomness is ubiquitous in the nervous system, from synaptic transmission to thermal noise causing neurons to spontaneously fire (White et al., 2000). When a perfectly critical network experiences noise, it moves away from the critical point in very specific ways. Intuitively, when the probability of spontaneous activity in neurons, *p*_*s*_, is increased, formerly distinct avalanches are concatenated (Figure 3) as shown in Williams-Garćıa et al. (2014). This increases the number of large avalanches, which decreases the slope of the distribution, and thus reduces the effective exponent. This can account for movement along the γ-scaling line toward the origin, as shown in Figure 4. This contrasts with what is expected to happen exactly at the critical point, where only one set of exponents should be found—a single point that does not move on the line—since that point (together with other exponents) represents a single universality class (Nishimori and Ortiz, 2011). Thus, moving exponents indicate that the network is not critical at all. But if the exponents move along the line, then the network is as close to critical as it could be—this is the origin of the term quasicriticality.

It is important to note that this phenomenon of avalanche concatenation will radically change the value of σ as traditionally measured, due to absence of separation of timescales between driving and relaxation processes. Hence, we use the branching parameter κ, the largest eigenvalue of the connectivity matrix, as the control parameter that tunes the system close to the peak of susceptibility. Note that κ can be considered to be the theoretical branching ratio, while σ is the empirical one measured without distinguishing concatenated avalanches, and in the critical case when *p*_*s*_ = 0 both will be unity. This parameter, and its relation to the branching ratio σ, is explained in Section 3.

**Figure 5:**
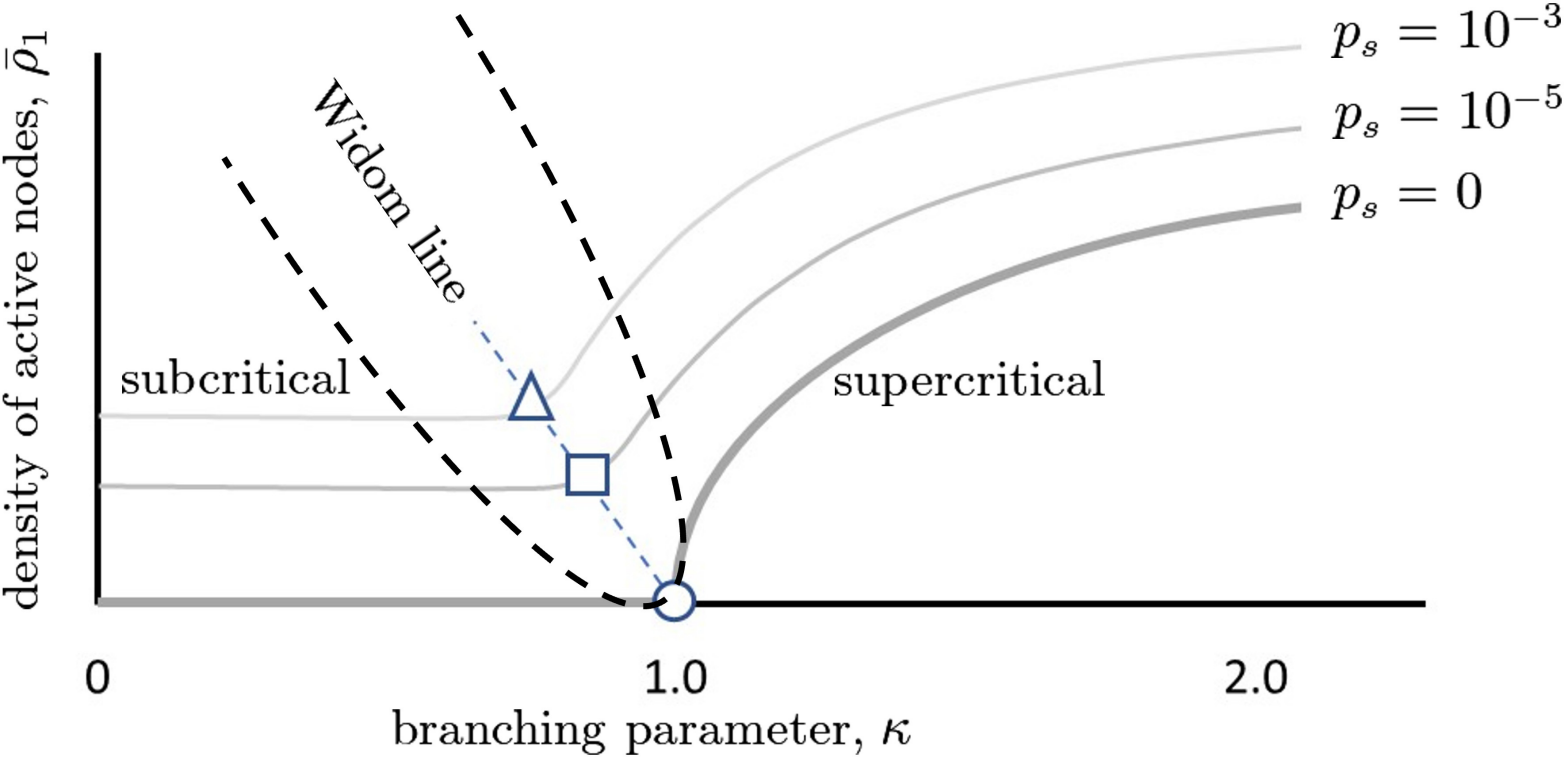
Phase diagram illustrating quasicriticality and the Widom line. When *p*_*s*_ = 0, there is an inactive phase to the left of the critical point (κ = 1 = σ) and an active phase to the right of it. The critical point is given by the circle. The thick gray curve shows how the fraction of active neurons (y-axis) varies as the control parameter κ is increased. As *p*_*s*_ is increased (lighter gray curves), this curve is shifted vertically and to the left. The points at which susceptibility will be maximal for each value of *p*_*s*_ are given by the square and the circle. While the network will not be critical at these points, it will be quasicritical (quasicritical region is enclosed with a parabolic black dashed line). This means that susceptibility will be maximal for that level of spontaneous activity. The dashed line joining these optimal points is called the Widom line. Note that the branching parameters at which maximum susceptibility occurs are now shifted to the left. We can also see that there are three main regions divided by the Widom line, the subcritical and supercritical ones. This figure schematically depicts predictions of a quasicritical network model (Williams-Garćıa et al., 2014) which have recently been corroborated with data from spiking networks (Fosque et al., 2021).

An increase in *p*_*s*_ has other effects as well. As there is more random activity, mutual information between inputs and outputs will be reduced; this is like experiencing static in a phone call and being less able to hear the speaker. Moreover, because the network will be less responsive to slight changes in the inputs, the susceptibility of the network will also decrease with increased *p*_*s*_ (Figure 4). To better illustrate, we draw a non-equilibrium phase diagram of the system (Figure 5) using the branching parameter κ as the control parameter. When *p*_*s*_ = 0, the critical point occurs at κ = 1 and there are two distinct phases: the inactive subcritical phase (κ < 1) and active supercritical phase (κ>1). When *p*_*s*_ is non-zero, however, the phase transition is replaced by a crossover region between less active and more active network states; there is always some network activity, even in the less active states. This crossover region is in fact the quasicritical region and contains the peak susceptibility for a given value of *p*_*s*_. The values of κ at which the susceptibility peaks for each value of *p*_*s*_ are themselves connected by the (dynamical) Widom line.

With large changes in *p*_*s*_, a network may need to “move” back to the quasicritical region by adjusting κ. As shown in Fosque et al. (2021), there is a correspondence between this “adjustment” and “movement” along the γ-scaling line, due to the change in the values of effective exponents ${\tilde{\tau }}_{T}$ and ${\tilde{\tau }}_{S}$ as *p*_*s*_ is changed. This is the underlying mechanism responsible for the movement of exponents along the γ-scaling line in Figures 2, 4. This picture of moving exponents fits well with recent reports that the exponents of a given animal can move along this exponent relation line over time (Fontenele et al., 2019) or after learning (Ma et al., 2020). Recent experiments with cortical slice cultures are also consistent with this framework (Fosque et al., 2021). Thus, although a quasicritical network is not at the critical point it is, in the sense described above, “close enough” to still enjoy improved information processing compared to networks that are arbitrarily away from the critical point (Helias, 2021). With this, we hypothesize that human MEG data will also fall along the γ-scaling line. Most importantly, the position along the γ-scaling line may reveal useful information about patient health and age. In this paper, we take the first steps toward exploring this idea. Can the position along the γ-scaling line be linked with basic patient information like age, gender, or sensitivity to new inputs?

## 3. Methods

### 3.1. Data and participants

We analyzed resting-state MEG data of 604 participants (304 male and 300 females) aged 18–88 years old provided in the Cam-CAN database. For each subject, temporal Signal Space Separation (tSSS, MaxFilter 2.2, Elekta Neuromag, Oy, Helsinki, Finland) was applied to remove noise from external source and from HPI coil. The sampling frequency of recorded data was 1 kHz with a high-pass filter of 0.03 Hz. Independent component analysis had been performed by Cam-CAN to exclude signal components associated with eye movements. The resting-state recordings were each at least 8 min and 40 s in duration. The MEG sensor array consisted of 306-channel Elekta Neuromag Vectorview (102 magnetometers and 204 planar gradiometers). We analyzed the magnetometer sensors only- although we have analyzed all directions, the magnetometer time-series provided better and more pronounced avalanche distributions. This gave us as a result 102 channels of time-series activity for each participant. More details about the data acquisition pipeline can be found in Taylor et al. (2017). Computations were carried out using MATLAB (R2020a, The Mathworks Natick, MA), and the Python programming language (Python Software Foundation. Python Language Reference, version 3.8. available at https://www.python.org/), GNU Parallel (Tange, 2011).

### 3.2. Extracting neuronal avalanches from MEG sensor activity

In MEG data, each channel's activity is presented in the form of a continuous-time series. The amplitudes, positive or negative, of these time-series determine the level of neural activity in the corresponding channel. To determine the significant, active, events we set a threshold separating relevant activity from background noise. In this way, we map to a discrete-time series. The method used amounts to:

- Let *N* = 102 be the total number of nodes/channels per subject, and *z*_*i*_ the state of the node *i*, *i* = 1, ⋯ , *N*. A node can have only one of two possible states *z*_*i*_(*t*)∈{0, 1} at a given time *t*.
- To determine the state of the node *z*_*i*_(*t*) from MEG data, we first subtract the mean activity and divide it by the standard deviation (SD). We then threshold the resulting time series so that events, positive or negative, that surpass the threshold level represent the active *z*_*i*_(*t*_*n*_) = 1 state while the rest is considered inactive, i.e., *z*_*i*_(*t*) = 0, for times *t*≠*t*_*n*_. We adjust the threshold by following the same procedure as in Dehghani et al. (2012) and Jannesari et al. (2020).

We found that a threshold much lower than 3 SD led to the detection of many noisy events, and 3 SD showed to be optimal for most subjects. This discretization map is similar to the one used in previous works (Shriki et al., 2013; Jannesari et al., 2020).

### 3.3. Effective critical exponents on the γ-scaling line

We have seen in previous work (Fosque et al., 2021) that the effective avalanche distribution exponents of a network with different parameters and noise will move along the γ-scaling line as long as the system lies in the quasicritical region. In this work, we explore the potential relation between the position of exponents on the γ-scaling line and the age and gender of the subjects. We thus need a way to project the pair of effective exponents (${\tilde{\tau }}_{S},{\tilde{\tau }}_{T}$), defining a point, onto the γ-scaling line to quantitatively asses its position along the line.

**Figure 6:**
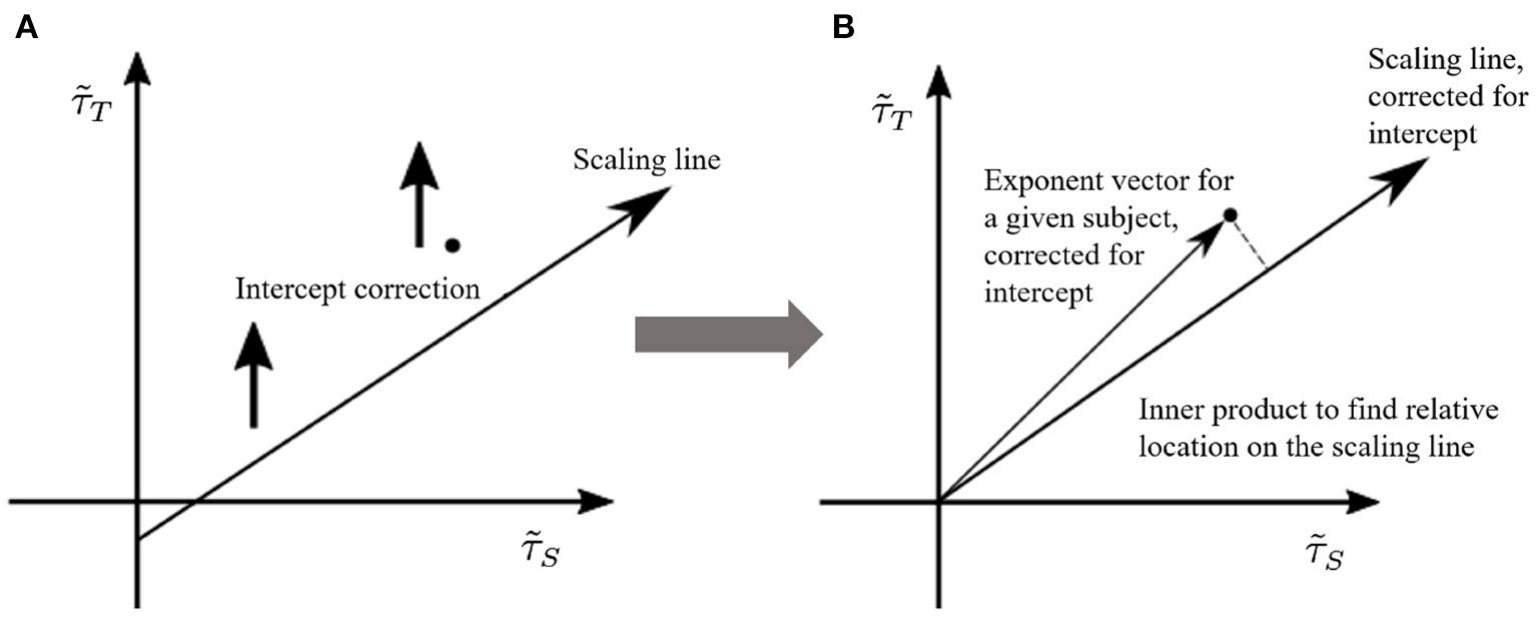
Pairs of size and duration effective exponents for each subject are transformed to a normalized value between 0 and 1. We call this measure the “position on the γ-scaling line.” This measure is obtained by **(A)** shifting the γ-scaling line, and **(B)** projecting each subject's duration-size vector onto the shifted γ-scaling line. The largest projection represents the value of 1.

To this end, once the γ-scaling line is determined, we consider the following projection method (see Figure 6):

- Shift vertically all points along the ${\tilde{\tau }}_{T}$ axis, so that the γ-scaling line intercepts the origin (0, 0). Note that we are only interested in the relative position on the line, so shifting all points does not affect the results.
- Project the vectors, defined from the origin to the shifted points (${\tilde{\tau }}_{S},{\tilde{\tau }}_{T}$), onto the new γ-scaling line as indicated in Figure 6B.
- Choose the projected point with largest distance from the origin on the shifted γ-scaling line. This distance will be defined as 1.
- Re-scale all other projected points by the maximal distance, so that all distances fall in the interval [0, 1].

Consequently, how far from the origin a given subject's point lies on the γ-scaling line provides a measure whose magnitude may correlate to age and/or gender, thus providing a biomarker.

### 3.4. Determining branching ratio σ and susceptibility χ

We used a recently-developed multi-step regression algorithm (MR. Estimator, Spitzner et al., 2021) to calculate the branching ratios, σ, of each subject. This algorithm allows to study subsampled time series and obtain the approximate, empirical, branching ratio σ.

We also calculated the local time fluctuation (LTF) as described in Fosque et al. (2021), which is similar to the coefficient of variation. The LTF measures the temporal fluctuations of the density of active nodes, or in a more colloquial sense, the variability of firing rate that the network displays in a given time interval. One starts by calculating the density of active nodes in the network at every time step,


$$\begin{array}{l} \rho_{1}\left(t\right)=\frac{1}{N}\overset{N}{\underset{i=1}{\sum }}\delta_{z_{i}\left(t\right),1}, \end{array}$$

where *N* is the total number of nodes, *z*_*i*_(*t*) is the state of node *i* at time *t*, and δ_*z*_*i*_(*t*), 1_ is 1 if *z*_*i*_(*t*) = 1 and zero otherwise. Then, we calculate the average firing rate for each subject by averaging the activity density over the total recording time *T*,


$${\langle \rho_{1}\left(t\right)\rangle }_{T}=\frac{1}{T}\overset{T}{\underset{t=1}{\sum }}\rho_{1}\left(t\right).$$

Notice that this definition of “firing rate” can never be greater than one because we are considering the fraction of population active at time *t*. We can also calculate the (dynamical) susceptibility defined as


(2) $$\begin{array}{l} \chi =N\left[{\langle \rho_{1}^{2}\left(t\right)\rangle }_{T}-{\langle \rho_{1}\left(t\right)\rangle }_{T}^{2}\right], \end{array}$$

where ${\langle \rho_{1}^{2}\left(t\right)\rangle }_{T}=\frac{1}{T}\overset{T}{\underset{t=1}{\sum }}\rho_{1}^{2}\left(t\right)$. Once the susceptibility, χ, and average firing rate, 〈_ρ_1_(*t*)〉*T*_, have been obtained one can calculate the LTF,


(3) $$\begin{array}{l} \text{LTF}=\frac{1}{{\langle \rho_{1}\left(t\right)\rangle }_{T}}\sqrt{\frac{\chi }{N}}. \end{array}$$

As seen from this Equation (3), if the standard deviation in average activity, $\sqrt{\chi }$, is large compared to the average firing rate, then LTF>1, which translates into a bursting activity, whereas whenever LTF < 1 the activity becomes more regular.

We also calculated the variance of avalanche sizes, *S*, for each subject,


(4) $$\begin{array}{l} \text{var}\left(S\right)=\langle S^{2}\rangle -{\langle S\rangle }^{2}, \end{array}$$

where $\langle S^{\nu }\rangle =\frac{1}{N_{av}}\overset{N_{av}}{\underset{j=1}{\sum }}S_{j}^{\nu }$ is the average of avalanche sizes to power ν(= 1, 2), *S*_*j*_ is the size of avalanche number *j*, and *N*_*av*_ is the total number of avalanches found in the given subject.

### 3.5. CBM: A minimal neural network model

Computational models are often extremely helpful in interpreting data and building intuitions about different mechanisms in nature. In neuroscience different models are proposed to mimic a variety of dynamical processes involving neurons. We are interested in understanding the collective behavior of a large number of neurons. Here, the computational neuroscience community is divided into two major camps: rate-type and spike-type models. For statistical analysis of avalanches, discrete-time spike models are more appropriate. In addition, our neuronal network is interacting spatially and temporally, that is, the state of a neuron will depend on the state of its connected neighbors at a previous time. Since the transmission of information between connected neurons is intrinsically probabilistic, the model needs to be stochastic. These are known as probabilistic cellular automata models.

While more biologically detailed models of cortical neural networks have been used to simulate nearly critical behavior (Markram et al., 2015; Del Papa et al., 2017), we did not take that approach. Here we were motivated by an extension of the principle of universality which can be invoked when systems operate near criticality (in particular, at the Widom line). Briefly, universality states that when the behavior of a system is scale-free, then its dynamics are similar across many scales; models therefore are not rooted in the details found at any particular scale. Rather, simple generic principles operate across scales, leading to correspondingly simple, conceptually-based models (Sethna et al., 2001; Beggs, 2022). Motivated by this, we employ a computational model based on our previous work (Williams-Garćıa et al., 2014; Fosque et al., 2021). Our cortical branching model (CBM), a probabilistic cellular automaton, makes use of the branching process to characterize avalanches and the spread of information across the network (Williams-Garćıa et al., 2014). The CBM is an elementary minimal model of cortex dynamics that encapsulates many of the experimentally relevant collective phenomena of neural networks. Although this model includes only excitatory nodes and fixed delays at 1 time-step, it reproduced relevant characteristics of biological neural networks such as avalanche distributions and raster plots seen experimentally in cortical slice networks (Beggs and Plenz, 2003; Haldeman and Beggs, 2005). We let each neural node have the same small probability *p*_*s*_ of spontaneous activation, mimicking the effect of external noise. Note that this model can also be used to obtain critical dynamics by letting *p*_*s*_ = 0 and κ = 1. The CBM is consistent with the organizing principle of quasicriticality but clearly many other models satisfy such principle ( including an extension of the CBM that incorporates inhibitory and rich club nodes; Weerawongphrom et al., 2022).

In this work, CBM simulations consisted of fully connected random networks, i.e., there is at least one path connecting any two nodes in the network. These networks had *N* = 256 nodes, each having a number of incoming neighbors *k*_*in*_ = 5, a connectivity matrix with weights *P*_*ij*_, and a fixed refractory period τ_*r*_ = 1 during which the node is not able to activate. Each node can be activated in two ways. First, it could be driven by its incoming neighbors. Second, it could activate spontaneously due to the external noise with probability $p_{s}=10^{-3}$. We will describe each of these next.

#### 3.5.1. Driven activity

Each connection from node *i* to node *j* has a weight *P*_*ij*_ = κ*p*_*n*_*ij*__ indicating the probability of transmission of activation *p*_*n*_*ij*__, 0 ≤ *p*_*n*_*ij*__ ≤ 1, randomly chosen from a weighting function (described below). The label *n*_*ij*_∈{1, ..., *k*_*in*_} ranks each neighbor connection coming from neighbor *i* to target node *j* by strength, e.g., *n*_*ij*_ = 1 corresponds to the strongest connection inbound at node *j*. The sum of probabilities emanating from each node *i* adds to one:


$$1=\overset{k_{in}}{\underset{i=1}{\sum }}p_{n_{ij}}.$$

Note that, when the network is tuned to be critical, the sum of incoming probabilities is the branching ratio σ by construction. We incorporate the branching parameter κ to modulate the state of the network. The relation between the branching ratio σ and the branching parameter κ is explained below. Node *j* at time step *t*+1 becomes active if node *i* in the previous time step *t* was active and the connection between them transmitted. A connection from *i* to *j* transmits if rand ≤ *P*_*ij*_, where *rand* is a uniformly distributed random number drawn from the interval [0, 1].

Processing nodes update at each time step to simulate the propagation of activity through the network. After becoming active, a node would become inactive, or refractory, for the next τ_*r*_ = 1 time steps.

#### 3.5.2. Branching

The branching parameter κ plays the role of the control parameter in the CBM, similar to temperature in the Ising model. This parameter places the system in or out of the quasicritical region. In the critical case, when *p*_*s*_ = 0, κ = 1 would put the system in the critical region. However, in the quasicritical case κ will have different values depending on the external noise *p*_*s*_ and other parameters of the network.

#### 3.5.3. Spontaneous activity

In addition to driven activity from neighbors, each node has a small probability, given by *p*_*s*_, of becoming spontaneously active at any time step. The network can only be critical when *p*_*s*_ = 0. As *p*_*s*_ increases, the network activity moves into the quasicritical region.

#### 3.5.4. Weighting function

As many studies report weight distributions with a nearly exponential form (Brunel et al., 2004; Barbour et al., 2007; Chen et al., 2010), we adopt a weighting function where transmission probabilities *p*_*n*_*ij*__ are determined by the following equation


$$p_{n_{ij}}=\frac{e^{-Bn_{ij}}}{\sum_{n=1}^{k_{in}}e^{-Bn}},$$

where *i*'s are neighbors to the target *j* node. When the bias exponent *B*≥0 equals zero, the distribution is homogeneous; as *B* increases, the distribution of *p*_*n*_*ij*__ values becomes increasingly inhomogeneous with few strong connections and many weak ones. Because data suggests that connection strengths become increasingly skewed with age, we wanted to be able to capture this in the model.

**Figure 7:**
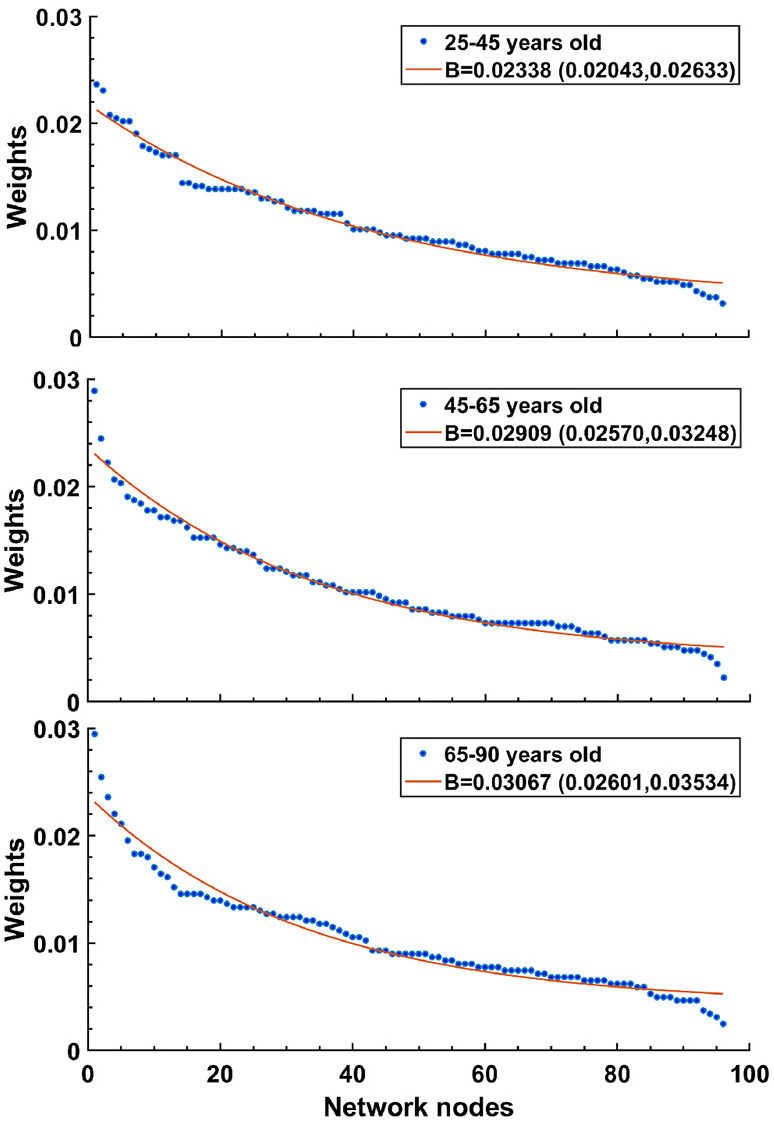
Human data suggests that exponential decay of connection strengths becomes steeper with age. Figure adapted from Otte et al. (2015). Note that oldest age group **(bottom)** shows the sharpest drop, while the youngest age group **(top)** has the shallowest curve. For details, see text.

We increased *B* to simulate the changes in connections reported in aging brains. Our justification for this was motivated by the results of Otte et al. (2015), who looked at connection strengths in human patients as they aged (see Figure 7). There, the density of fiber bundles was assessed by Diffusion Weighted Imaging (DWI). The entire cortical mantle was parcellated into regions (nodes) and the strength of each node was taken as the sum of connection strengths into and out of a region. When average node strengths were arranged in descending order and then separated by age groups, it revealed that curves from older patients dropped more steeply than for younger patients. The best fit exponential curves for the three age groups revealed a trend, where the exponents became larger for older cohorts. For ages 25–45: 0.02338 (0.02043, 0.02633), ages 45–65: 0.02909 (0.0257, 0.03248), and ages 65–90: 0.03067 (0.02601, 0.03534); figures given as mean value, followed by 95% confidence limits. It is important to stress that in our CBM we effectively mimic the reduction in the number of connections by skewing the weights of the connections while keeping *k*_in_ fixed. We found that random removal of connections in a simulation results in an increase in the bias parameter, *B*. This is because most connections have relatively weak strengths, so random removal preferentially reduces weak strengths, increasing the skew, and therefore *B*.

## 4. Results

### 4.1. γ-Scaling line results

**Figure 8:**
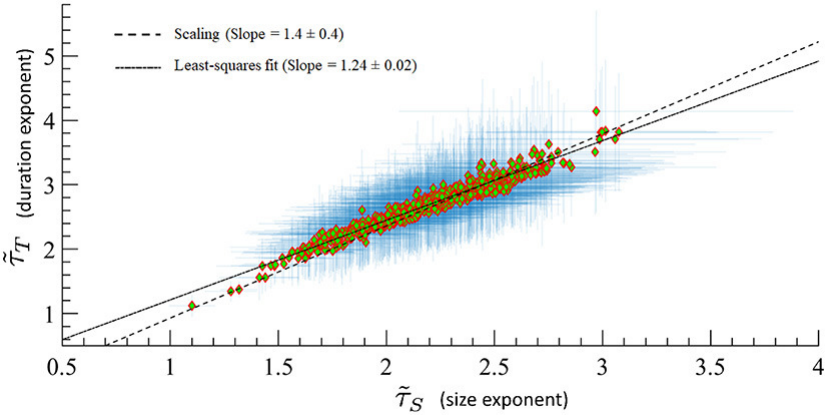
MEG data from 566 human subjects on the γ-scaling line with ages ranging from 18 to 88 years old. Error bars are shown in blue. A least-squares fit of exponent pairs is also shown in dashed line, and gives a least-squares fit of γ_lsf_ = 1.24 ± 0.02. Solid line indicates the average scaling slope, $\langle \frac{{\tilde{\tau }}_{T}-1}{{\tilde{\tau }}_{S}-1}\rangle =1.4\pm 0.4$. The scaling slope is the average of scaling fraction across subjects.

A major prediction of quasicriticality is that human MEG data should organize along a γ-scaling line just as has been seen previously with animal's spiking data (Fontenele et al., 2019; Fosque et al., 2021). To test this, we calculated effective exponents from the avalanche distributions of the included MEG data and found, as predicted, that all these exponents are distributed along the γ-scaling line. The average γ exponent for all datasets is 〈γ〉 = 1.07 ± 0.03, and the average scaling $\langle \frac{{\tilde{\tau }}_{T}-1}{{\tilde{\tau }}_{S}-1}\rangle =1.4\pm 0.4$. Recall that γ is obtained from the power-law relation between the average avalanche size distribution 〈*S*〉 and the avalanche duration *T*. These results are consistent within statistical error bars, and we need to remember that the scaling fraction is sensitively dependent upon errors of the measured effective exponents, so larger errors are to be expected. Figure 8 shows all datasets on the γ-scaling line with their corresponding error bars. These results show a similar characteristic distribution of effective exponents as in Fontenele et al. (2019) and Fosque et al. (2021) although with a slightly different slope. Hence, we see that these results appear to be consistent with the organizing principle of quasicriticality.

**Figure 9:**
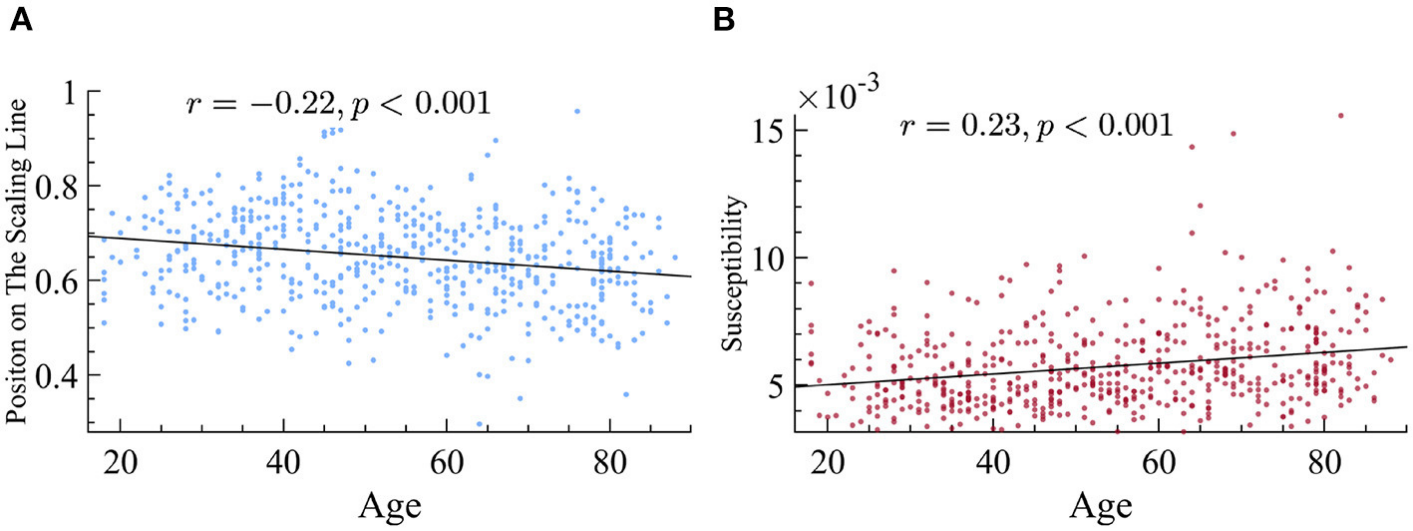
Older subjects display smaller exponents and higher susceptibility. **(A)** Older subjects have exponents that fall lower on the γ-scaling line. We found that there is a significant negative correlation between age and the magnitude of time and size exponents in our dataset. **(B)** Older subjects have higher dynamical susceptibility. There is a significant correlation between age and susceptibility.

After confirming that the data followed the principle of quasicriticality, we next sought to find out if the position on the γ-scaling line could reveal information about the patient population. We first looked at the differences between age groups with respect to their exponents. We used the distance measure explained earlier and found that there is a small but significant negative correlation between age and the position on the line. More specifically, being older is found to be negatively correlated with the magnitude of the duration and size exponents (Figure 9) and therefore, a lower location on our previously defined position on the γ-scaling line.

**Figure 10:**
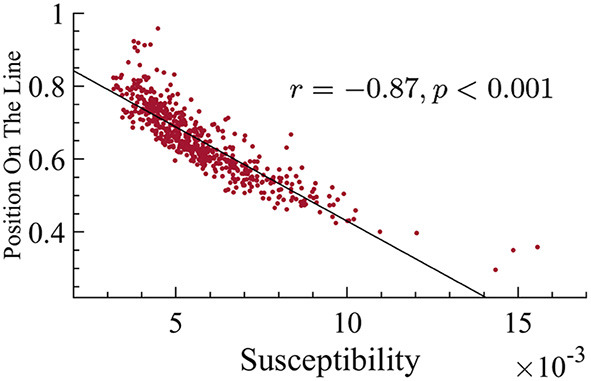
There is a negative correlation between position on the line and susceptibility. This correlation suggests that subjects with smaller exponents have higher susceptibility (correlation *r* = −0.87, *p* < 0.001).

Another prediction of quasicriticality is that when *p*_*s*_ increases, susceptibility decreases (as long as the system is near the peak of maximum susceptibility), and exponents get smaller, which translates into a position closer to the origin on the γ-scaling line. Hence, after finding the differences in position on the γ-scaling line with respect to age, and the relation with their exponents, we decided to analyze their susceptibility. We found that there is a strong and negative correlation between susceptibility and position on the line. In other words, subjects with smaller effective exponents show higher susceptibility in their MEG data, see Figure 10 for details. Since older subjects have lower position on the line, this means that the older subjects also have higher dynamical susceptibility (see Figure 9). This result may seem to contradict the prediction of quasicriticality, but recall that the susceptibility depends on multiple parameters of the network. We will show below a potential parameter that may be causing this trend on the γ-scaling line.

**Figure 11:**
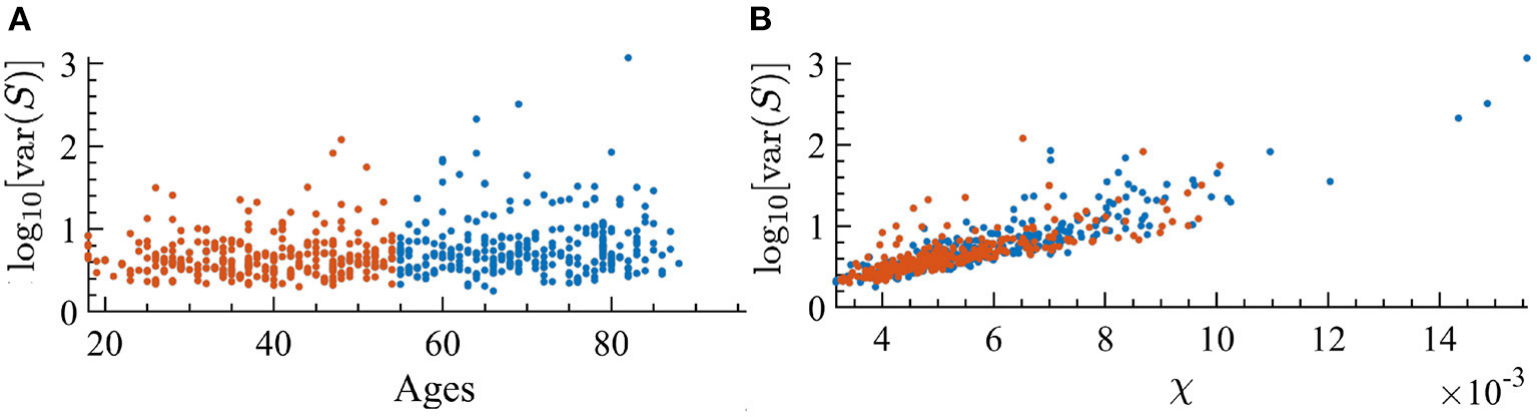
Variance of the avalanche size, var(*S*), for each individual; orange indicates the youngest half and blue the oldest half. **(A)** var(*S*) as a function of the age of individuals. We find a correlation with age of *r* = 0.213 with *p* < 0.001 in the log-linear scale. **(B)** var(*S*) vs. susceptibility, χ, for each subject. We find a correlation with susceptibility of *r* = 0.8449 with *p* < 0.001 in the log-linear scale.

It is well-known that as external noise is decreased, i.e., approaching criticality, the variance of avalanche sizes, var(*S*), will diverge (Pinto and Muñoz, 2011). Since the susceptibility displays similar behavior, we are interested in looking at the relation between var(*S*) and dynamical susceptibility χ across ages of individuals. In Figure 11, one can see that there is indeed a clear correspondence between them. Furthermore, as age increases, there is a correspondence with an increase in the variance of avalanche sizes.

**Figure 12:**
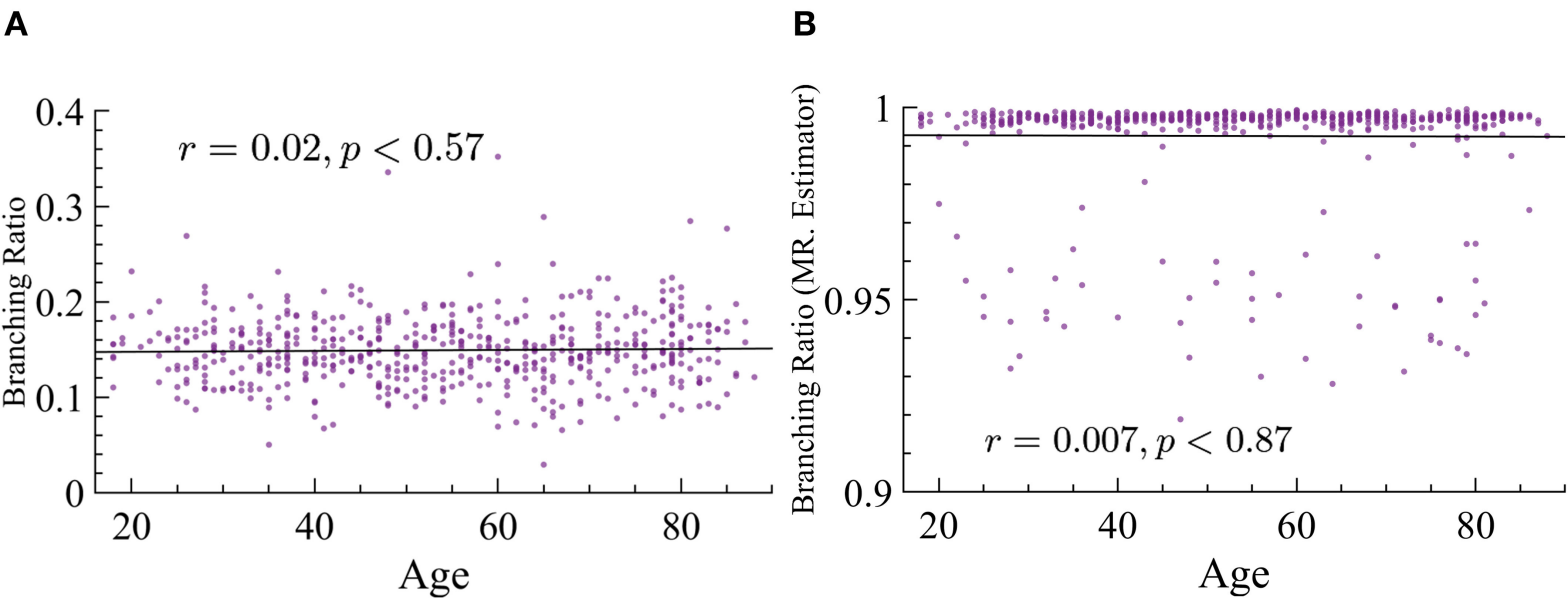
Branching ratio does not significantly change with age. **(A)** Naive method: *r* = 0.02, *p*≈0.57. **(B)** MR. Estimator : *r* = −0.007, *p*≈0.87.

As stated in the Background section, there is an important relation between susceptibility and branching parameter κ. This relation states that the peak of maximum susceptibility will move toward lower values of the branching parameter as *p*_*s*_ is increased. Our analysis of susceptibility showed that older subjects have higher values of susceptibility than younger ones. Since we cannot extract the parameter κ from this data, and we know that there is a relation between κ and σ, we determine σ instead experimentally. However, one of the most striking results is the fact that the branching ratios did not significantly change across age groups. We used both MR. Estimator and naive methods and find that, regardless, this parameter does not change with age, see Figure 12 for more details.

**Figure 13:**
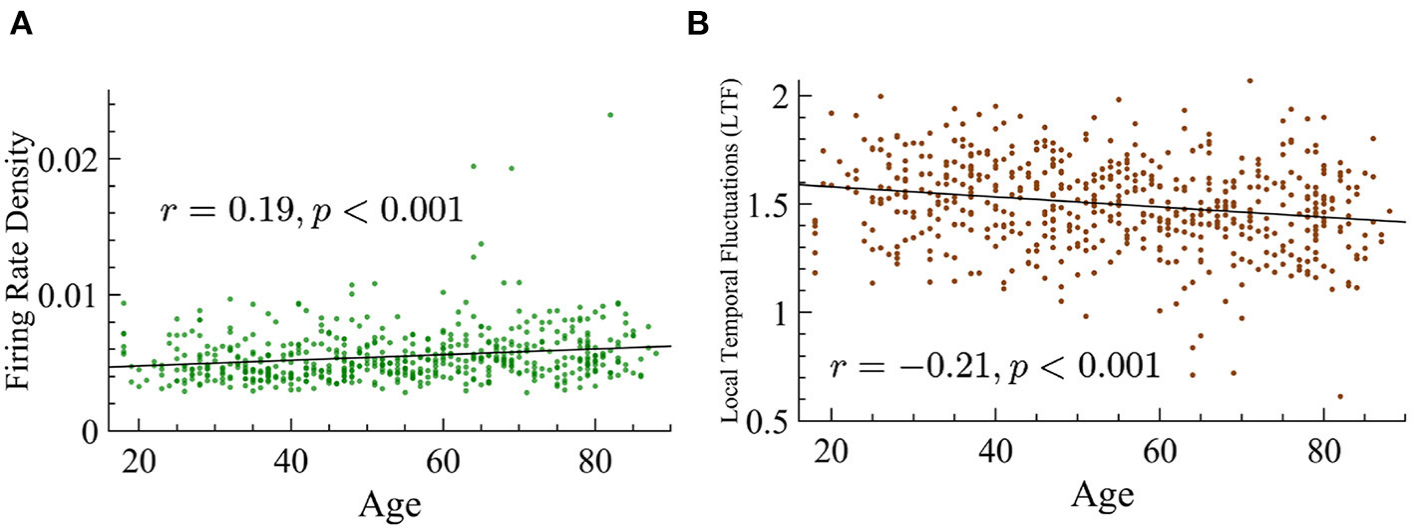
Age positively correlates with firing rate density and shows a negative correlation with LTF. **(A)** Firing rate density vs. age shows a significant positive correlation (*r* = 0.19, *p* < 0.001). The statistical significance of this relationship is not dependent on the three outlier values that are above 0.015. **(B)** LTF vs. age shows a significant negative correlation (*r* = −0.21, *p* < 0.001).

Quasicriticality also predicts that with increasing external noise there will be an increase of avalanche concatenation, which in turn would result in smaller exponents. We mentioned before that the LTF, as the coefficient of variation, measures the level of variability in activity in the network. Similarly, the firing rate of a network can give clues on the level of activity in the subject given that an increase in the firing rate increases the probability of larger avalanches. Hence, we also analyzed the variability in activity across the different age groups by calculating the LTF and the firing rate density for each subject as defined in Methods. LTF is found to be reduced with age, and the firing rate density is found to increase with age. Although we see variability around the average, the trend is very consistent, *p* < 0.001 in both cases, see Figure 13 for more details. It is important to remember that an increase in external noise will result in a lower value of LTF, but a low value of LTF does not necessarily imply an increase in *p*_*s*_ since there are other parameters that can influence this variability.

**Figure 14:**
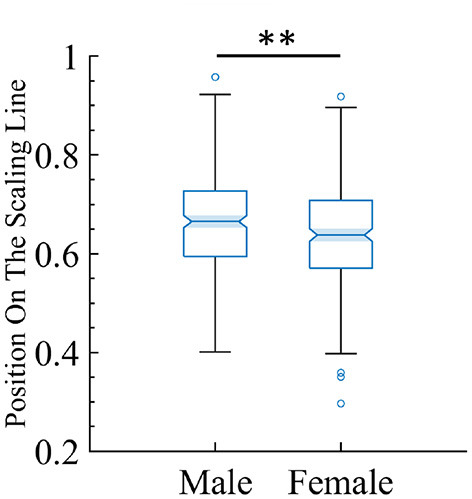
There is a small but statistically significant difference in position on the line between male and female subjects. The box plot shows the distribution of the female and male subjects' data and where they land on the line. For female subjects (mean = 0.64, SD = 0.10), while for male subjects (mean = 0.66, SD = 0.09). The two-tailed t-test gives *t* = −2.97 and *p* < 0.01 (indicated by the two stars).

One of the goals of this study is to find potential biomarkers by using the framework of quasicriticality. Thus, given the vast amount of information contained in this large MEG dataset, we decided to see if we could use our framework to predict gender from the data. We used the position on the γ-scaling line measure to investigate whether the magnitude of the duration and size exponents are different between male and female subjects. Indeed, there is a significant difference, suggesting that this approach may hold promise as a biomarker. We found that male subjects have a slightly higher position magnitude compared to female subjects (see Figure 14).

### 4.2. CBM model fits the age related reduction in exponents

**Figure 15:**
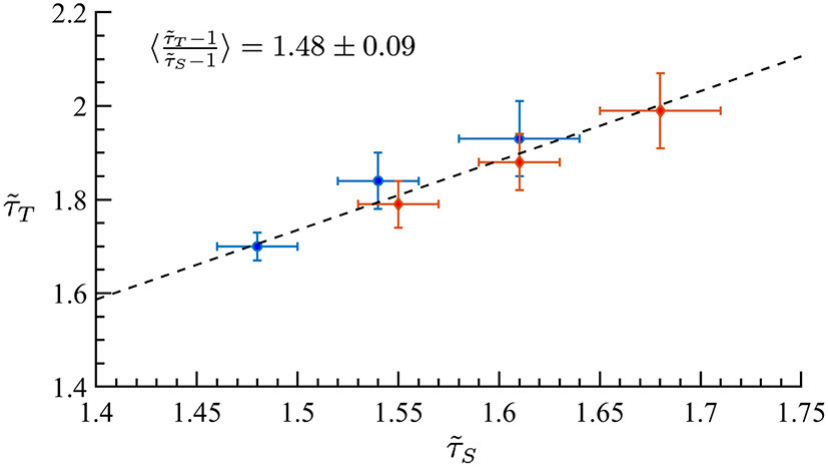
Simulation on the γ-scaling line. Probability of spontaneous activation $p_{s}=10^{-3}$, and bias parameter *B* = 0.6 (red diamonds) and *B* = 1.8 (blue circles). The three points for each bias represent three different κ values around the peak of maximum susceptibility taken from Tables 1, 2. We can see that as we increase the bias parameter, the exponents get smaller while increasing susceptibility.

Motivated by the lower exponent values among older subjects, we sought to create a model that can account for this experimental finding. As mentioned in Methods previous literature (Otte et al., 2015) indicates that natural/healthy aging involves the weakening of weak weights and enhancement of strong connections in functional connectivity. We ran simulations with our CBM with different weight distributions to mimic this trend. In our CBM the steepness of these distributions is characterized by the bias parameter *B*. We ran CBM simulations with bias parameter *B* = 0.6 and *B* = 1.8. Recall from Section 3, that a higher value of *B* means a more positively skewed distribution of weights while a lower value of *B* would correspond to a flatter distribution. We observed that simulations with a bias of *B* = 0.6 have a lower susceptibility peak but larger exponent values than the simulations with larger bias. These simulations were run under the same probability of spontaneous activations, $p_{s}=10^{-3}$, and on a network with 256 nodes, 5 incoming neighbors, and refractory period of one time step. These results indicate that making connectivity weights steeper reduces the size and duration exponents (Figure 15 and Tables 1, 2). These results from the CBM simulations fit the trend of the MEG experimental results where there is an overlap of exponents along the γ-scaling line whenever the system is around the peak of maximum susceptibility.

**Table 1:** Table of exponents from CBM simulations for bias *B* = 1.8 for κ-values around the peak of maximum susceptibility.

**κ**	**χ**	** ${\tilde{\tau }}_{S}$ **	** ${\tilde{\tau }}_{T}$ **	** $\frac{{\tilde{\tau }}_{T}-1}{{\tilde{\tau }}_{S}-1}$ **	**γ**	**log_10_[var(*S*)]**
1.06	0.9 ± 0.2	1.48 ± 0.02	1.70 ± 0.03	1.46 ± 0.03	1.52 ± 0.02	8.9
1.07	0.9 ± 0.1	1.54 ± 0.02	1.84 ± 0.06	1.56 ± 0.05	1.54 ± 0.02	9.3
1.08	0.9 ± 0.2	1.61 ± 0.03	1.93 ± 0.08	1.54 ± 0.07	1.52 ± 0.02	10.3

**Table 2:** Table of exponents from CBM simulations for bias *B* = 0.6 for κ-values around the peak of maximum susceptibility.

**κ**	**χ**	** ${\tilde{\tau }}_{S}$ **	** ${\tilde{\tau }}_{T}$ **	** $\frac{{\tilde{\tau }}_{T}-1}{{\tilde{\tau }}_{S}-1}$ **	**γ**	**log_10_[var(*S*)]**
1.11	0.44 ± 0.02	1.55 ± 0.02	1.79 ± 0.05	1.44 ± 0.04	1.53 ± 0.02	8.1
1.12	0.44 ± 0.01	1.61 ± 0.02	1.88 ± 0.06	1.44 ±0.05	1.53 ± 0.02	8.6
1.13	0.43 ± 0.02	1.68 ± 0.03	1.99 ± 0.08	1.46 ± 0.06	1.52 ± 0.02	8.9

Thus, our computational model can capture the trends observed in the data when it is supplied with similar connectivity parameters.

## 5. Discussion

We began this paper by suggesting that neural biomarkers could be based on quantities that are homeostatically regulated for health, like functional neuronal activity. The principle of quasicriticality predicts that neural networks will operate in a quasicritical region associated to a critical point, even in the face of perturbations, by moving along a dynamical scaling line. We hypothesized that a patient's position along this scaling line could therefore contain valuable health information about their relation to their “normal state” and various factors that might perturb them from it. Here we used a large MEG data set to conduct a first test to see if this quasicritical framework could show potential for developing biomarkers. Our first main finding was that the majority of MEG data indeed fell along the scaling line. The second main finding was that the position on this line was significantly related to the age of the subject and their gender. The third main finding was that the susceptibility of the subjects, akin to their sensitivity to new stimuli, was also significantly related to their position along the scaling line. To make sense of these findings, we employed a previously published network model, the cortical branching model. When the model was supplied with connectivity data that reflected the age of the patients, it qualitatively reproduced the results found in the data—this is our fourth main finding. Quasicriticality can thus offer a plausible explanation as to why older subjects become more susceptible to stimuli or noise as their distribution of neural connections become skewed with age. We suggest that the quasicritical framework therefore should be further investigated as a platform for developing biomarkers of neurological health.

The observation associated with the preservation of branching ratios is quite intriguing. We do not yet have a full theoretical understanding of the reason behind this *potential conservation law*. In quasicriticality, when the system reaches the peak of maximum susceptibility, it will fit a branching parameter κ, which when *p*_*s*_ → 0, κ = 1 = σ. The fact that σ remains stable may suggest a conservation law.

Previous work on human MEG data showed that it contained signatures of criticality in the form of avalanche distributions that followed power laws, both for spontaneous activity (Shriki et al., 2013) and stimulus-evoked activity (Arviv et al., 2015). The work by Arviv et al. (2015) even examined the scaling relation but did not find it to be well-satisfied. They speculated that this was because there were not enough stimulus-evoked data to conclusively evaluate its validity (Arviv et al., 2015). In the present work, we expanded on these pioneering results by exploring an MEG data set of spontaneous activity that was almost thirty times larger (*n* = 21 vs. *n* = 604) and, at the same time, we broaden the framework to that of quasicriticality, a more explanatory framework. Because of this, we were able to examine the scaling relation more fully, finding that it fit within experimental error for the vast majority of patients.

Many other groups have noted connections between criticality and neurological health. Broadly speaking, these works have used distance from the critical point, variously assessed by the branching ratio, the quality of the power laws/avalanche shape collapse, the extent of multifractality, or the degree to which the exponent relation is satisfied as the relevant variables. For example, this approach has been taken with respect to sleep and sleep deprivation (Meisel et al., 2013; Priesemann et al., 2014), epilepsy (Meisel et al., 2012, 2015; Arviv et al., 2016; Hagemann et al., 2021), hypoxia (Roberts et al., 2014), stroke (Rocha et al., 2022), schizophrenia (Alamian et al., 2022), and Alzheimer's disease (Jiang et al., 2018), to name a few. For overviews, see Massobrio et al. (2015), Zimmern (2020), and Fekete et al. (2021).

Although our present work shows a few similarities with these previous studies, we are the first, to our knowledge, to indicate how the position along the scaling line may be used as a potential biomarker. Strictly speaking, our work does not evaluate distance to the critical point, but rather how the system configures itself in exponent space as it continues to maintain its activity homeostatically in the quasicritical region close to the Widom line, i.e., the line of maximal susceptibility. More concretely, two patients could be equally close to the critical point by some measure but could still lie on different portions of the scaling line. This illustrates that we are taking a qualitatively different perspective from that of previous studies, one that could potentially reveal new information.

Because this work was only a first step toward using the principle of quasicriticality to develop biomarkers, it has room for improvements. First, the MEG recordings were rather brief, averaging about 9 min per subject, which curtailed the statistical power of our analyses. In the future, it would be better to have longer recordings (30–60 min) with more neuronal avalanches. Second, the MEG scanner used to produce this data set provided 102 time series. While this is state of the art, in the future we would like to increase the number of channels to enhance statistics. Third, in this work we used a data set that had only the most basic biological information like age and gender; this restricted the types of conclusions that we could draw. A data set that contained information about multiple health disorders like the degree of dementia, epilepsy, depression, or schizotypy would have allowed many dimensions of neurological health to be checked for relationships with the position of each subject in exponent space along the scaling line. These proposed improvements should be addressed in future work.

Very generally, the concept of criticality implies that a system must have a parameter that is precisely tuned to the critical point. In contrast, quasicriticality allows multiple parameters to interlock in specific ways to confine the system to a line or surface that could be considered approximately critical. Consistent with this, several studies have shown that as neural systems remain close to criticality, their avalanche distribution exponents vary in a peculiar way (Shew et al., 2015; Fontenele et al., 2019; Ma et al., 2020). Specifically, the exponent γ (for average size against duration) typically remains nearly fixed, while ${\tilde{\tau }}_{S}$ (for size) and ${\tilde{\tau }}_{T}$ (for duration) may vary but continue to nearly satisfy the exponent relation. The system is thus confined to move along a line. The fundamental reason behind a nearly constant γ and why ${\tilde{\tau }}_{S}$ and ${\tilde{\tau }}_{T}$ are the exponents that vary is not known; these are intriguing open questions.

But because of these facts, we are now able to explore how nearly critical combinations of ${\tilde{\tau }}_{S}$ and ${\tilde{\tau }}_{T}$ relate to biological features. In the present work, we made first steps by linking them to age, gender, and susceptibility. Now that this has been established, future work could investigate how these exponents relate to features of neurological disorders, as well as health features like resilience, creativity, or intelligence.

To illustrate the potential utility of this, let us consider two scenarios: (1) departures from normality, and (2) dynamics of recovery. For (1), individual patients may have a tendency to spend more time on a specific region of the scaling line. Physicians could track this yearly as an indicator of what is typical for that patient. Sudden departures from that region of the scaling line could then signal that the patient's brain is trying to compensate for some new neurological stress. The presence of this stress could be revealed in a yearly checkup by measuring the patient's exponents. For (2), just as a cardiologist may apply a difficult treadmill challenge to a patient to stress test their heart and watch its recovery, a neurologist could apply a cognitive challenge to a patient and watch how the exponents move along the scaling line to assess recovery. The dynamics of how the exponents move back toward their original position may reveal the strength of homeostasis in that patient.

## Data availability statement

The datasets presented in this study can be found in online repositories. The names of the repository/repositories and accession number(s) can be found in the article Methods section.

## Ethics statement

Ethical review and approval was not required for the study on human participants in accordance with the local legislation and institutional requirements. The patients/participants provided their written informed consent to participate in this study.

## Author contributions

LF performed the simulations and carried out the analysis of data and simulations. JB, LF, and AA presented the idea of connectivity and aging. MZ contributed with the MEG data and started the study. RW-G contributed with the CBM code. GO developed the theoretical formalism in relationship to quasicriticality. GO and JB developed the concepts and wrote the manuscript. All authors discussed the results of the analysis and read, commented, and contributed to the content of the paper.

## Funding

MZ was supported through funding from—IVADO postdoctoral fellowship program—PD-2019a-FQ-10, Montreal, CA. JB was supported through NSF grant 2123781 (subcontract to JB).

## Conflict of interest

Author MZ was employed by CGI. The remaining authors declare that the research was conducted in the absence of any commercial or financial relationships that could be construed as a potential conflict of interest.

## Publisher's note

All claims expressed in this article are solely those of the authors and do not necessarily represent those of their affiliated organizations, or those of the publisher, the editors and the reviewers. Any product that may be evaluated in this article, or claim that may be made by its manufacturer, is not guaranteed or endorsed by the publisher.
