# Supplementary material for: Quasicriticality explains variability of human neural dynamics across life span
Source: Front Comput Neurosci. 2022 Nov 30;16:1037550. doi: 10.3389/fncom.2022.1037550 (PMC9747757; doi:10.3389/fncom.2022.1037550)
Supplement: Supplementary file 1 [file Data_Sheet_1.PDF]

Supplementary Material

We present some supplementary material on avalanche distributions for the MEG data and CBM simulations used in the manuscript. To reduce noise, the size and duration distributions were logarithmically binned using a binning factor $a = 1.1$ as described in page 356 of Christensen and Moloney (2005).

1 AVALANCHE DISTRIBUTIONS FOR MEG DATA TEMPORALLY BINNED AT 4MS

Here we present an example of an exponent estimation for one subject. As can be seen in Fig. S1, the limited number of avalanches, approximately 26000, leads to fairly large errors. The reasons behind this is due to the short recording times and the fact that not all sources are captured in MEG recordings (due to orientational subsampling).

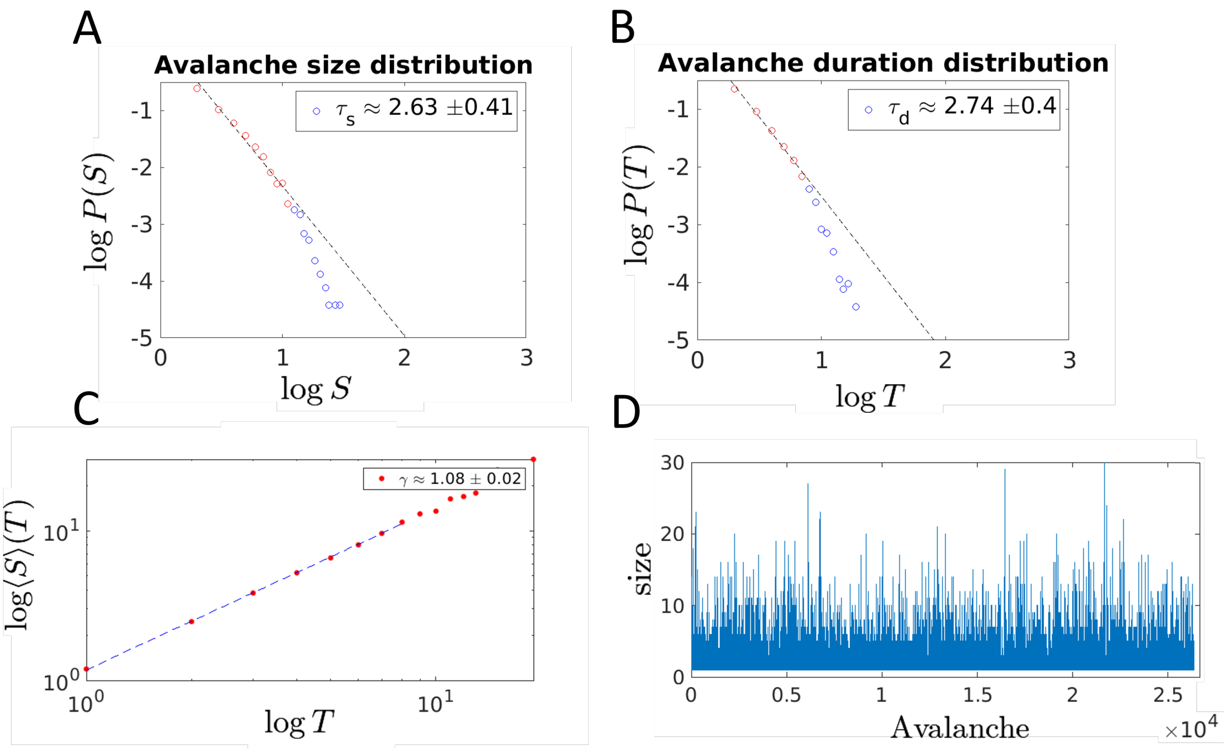

Figure S1. Avalanche distributions for a typical subject in the dataset. **A:** Size distribution. **B:** Duration distribution. **C:** Average size with respect to duration distribution, demonstrating Sethna scaling. **D:** Avalanche sizes with respect to avalanche id in time of appearance.

2 AVALANCHE DISTRIBUTIONS FROM CBM SIMULATIONS

Here we present an example of avalanche distributions from CBM simulations using a probability of spontaneous activation of $p_s = 10^{-3}$. In Fig. S2 we see the exponent estimation from the CBM simulation with $N = 256$, incoming neighbors $k_{\text{in}} = 5$, refractory period $\tau_r = 1$, bias $B = 1.8$, and branching parameter $\kappa = 1.08$ which is at the peak of maximum susceptibility, $\chi_{\max}$. In Fig. S3 we see that when the same system is a on the subcritical side of $\chi_{\max}$, the distribution resembles more what we see in the dataset in Fig. S1. Note that our simulations used a bigger system size to get better statistics, for our main interest

is to understand the trends along the scaling line. When the system size is the same as for the MEG data ($N = 100$), as shown in figure S4, we see that the exponents are a bit larger than in the previous figure ($N = 256$) under the same parameters.

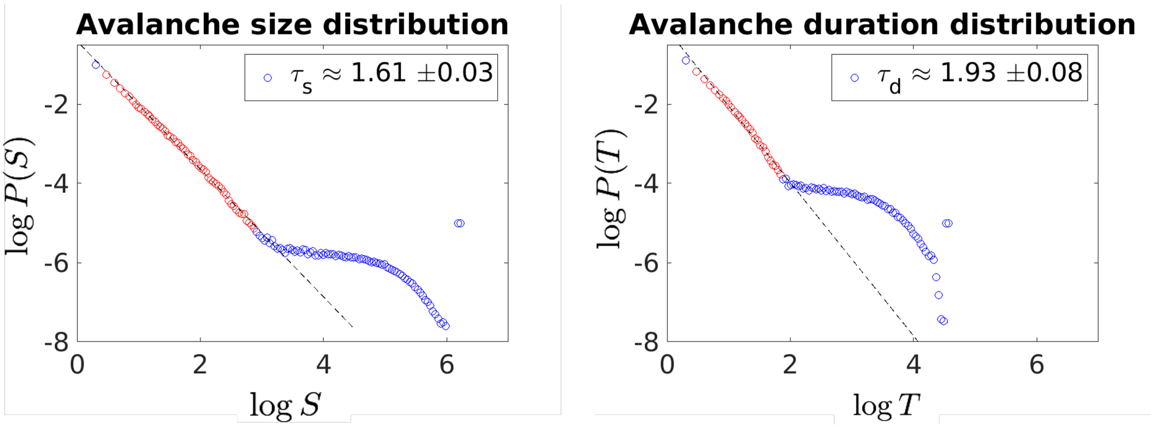

Figure S2. Avalanche distributions for $p_s = 10^{-3}$, $N = 256$, $\kappa = 1.08$, and $B = 1.8$. This corresponds to a system at the Widom line, the line of maximal dynamical susceptibility.

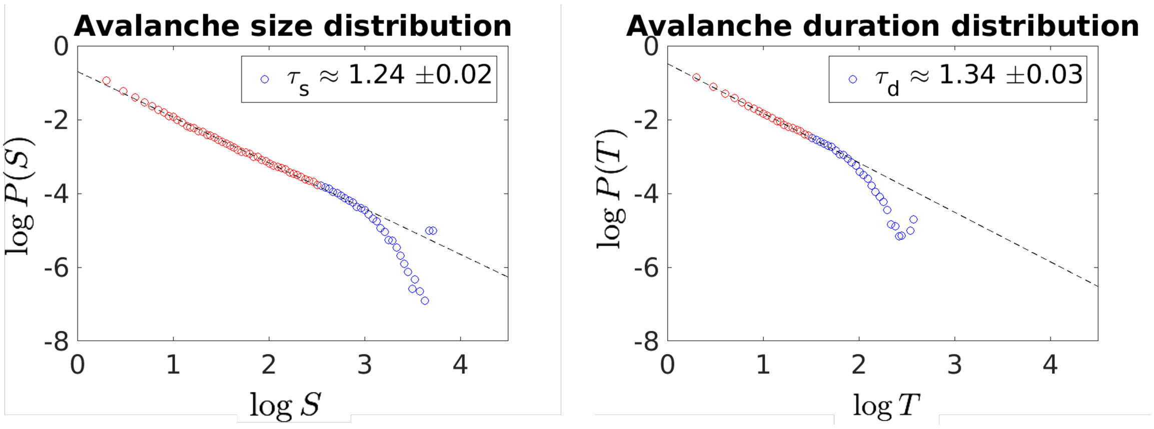

Figure S3. Avalanche distributions for $p_s = 10^{-3}$, $N = 256$, $\kappa = 0.97$, and $B = 1.8$. This corresponds to a system in the quasicriticality region, away from the Widom line, towards the subcritical regime.

REFERENCES

Christensen, K. and Moloney, N. (2005). *Complexity and Criticality* (Imperial College Press)

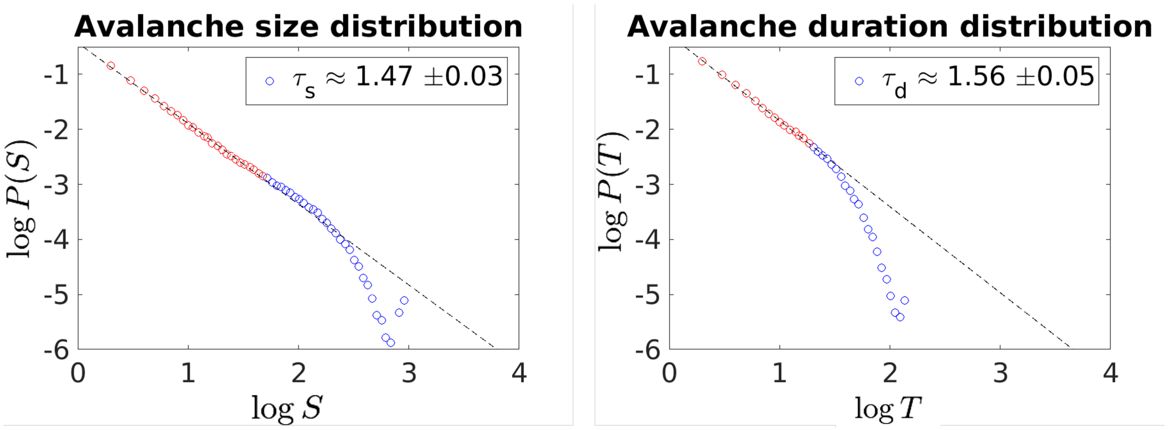

Figure S4. Avalanche distributions for $p_s = 10^{-3}$, $N = 100$, $\kappa = 0.97$, and $B = 1.8$. Similar to Fig. S3, but with same system size as found in MEG data, $N = 100$. This corresponds to a system in the quasicriticality region, away from the Widom line, towards the subcritical regime.
